# Toll-Like Receptor 4 Signaling in the Trabecular Meshwork

**DOI:** 10.3389/fcell.2022.936115

**Published:** 2022-07-15

**Authors:** Philip Mzyk, Humberto Hernandez, Thanh Le, Jose Ricardo Ramirez, Colleen M. McDowell

**Affiliations:** ^1^ University of Wisconsin-Madison, Madison, WI, United States; ^2^ University of Houston-Victoria, Victoria, TX, United States

**Keywords:** trabecular meshwork, TLR4, ECM, intraocular pressure, glaucoma

## Abstract

Primary open-angle glaucoma is one of the leading causes of blindness worldwide. With limited therapeutics targeting the pathogenesis at the trabecular meshwork (TM), there is a great need for identifying potential new targets. Recent evidence has implicated Toll-like receptor 4 (TLR4) and it is signaling pathway in augmenting the effects of transforming growth factor beta-2 (TGFβ2) and downstream extracellular matrix production. In this review, we examine the role of TLR4 signaling in the trabecular meshwork and the interplay between endogenous activators of TLR4 (damage-associated molecular patterns (DAMPs)), extracellular matrix (ECM), and the effect on intraocular pressure

## Introduction

Glaucoma is a progressive neurodegenerative disease and is the second leading cause of blindness worldwide, affecting over sixty million people (Kingman, 2004; Quigley and Broman, 2006). Primary open-angle glaucoma (POAG) is the most common form of the glaucoma’s and affects approximately fifty-two million people worldwide and more than 2.5 million in the United States (Friedman et al., 2004; Weinreb and Khaw, 2004; Weinreb et al., 2014; Lu et al., 2017; Zhang et al., 2021). Current therapies are supportive, with the aim to reduce intraocular pressure (IOP), a primary risk factor for glaucoma progression. IOP homeostasis is maintained by the rate at which aqueous humor (AH) is secreted by the ciliary epithelium, and how efficiently it is drained through the outflow pathways in the iridocorneal angle of the eye. Most of the outflow of AH drains through the conventional route of drainage, which is made up of the trabecular meshwork (TM) and Schlemm’s canal (Tamm, 2009). The TM is well known to be a critical tissue in AH drainage and imparts a normal resistance to AH outflow that becomes abnormally increased in glaucoma. The TM is a porous structure consisting of a series of fenestrated beams and sheets of extracellular matrix (ECM) covered with endothelial-like TM cells (Hogan et al., 1971; Vranka et al., 2015). The ECM of the TM is important in forming a fluid flow pathway for AH drainage (Gong et al., 1996; Morrison and Acott, 2003). Genes that are broadly categorized as regulating cell signaling comprise the highest percentage of genes that are upregulated in the TM when their homeostatic state is altered, such as during changes in IOP (Vittitow and Borrás, 2004). The ability of the TM to respond to the dynamic changes in IOP in a homeostatic state relies on the ECM remodeling capabilities of the TM (Keller et al., 2009). When this ability becomes impaired, IOP rises and can eventually lead to vision loss. While current drug and surgical interventions are in use, their efficacy is not always guaranteed or long lasting. Additionally, many of the current therapeutics aim to decrease the production of aqueous humor and are not able to target the key site of drainage impairment in glaucoma, which is the TM. While progress has been made in increasing the efficiency of humor drainage through the outflow pathway, such as with prostaglandins and Rho-kinase inhibitors, these therapies address only a small fraction of the mechanisms used by the TM to exert its function and thus more treatment options are needed. Therefore, much effort is being conducted into understanding the molecular pathways of the TM and how they are altered during glaucoma.

Increases in outflow resistance through the TM can be contributed to multiple factors, such as actin cytoskeletal rearrangement in the TM and changes to the inner wall endothelium of Schlemm’s canal (Stamer and Acott, 2012; Vahabikashi et al., 2019). Additionally, the ECM composition of the outer most layer of the TM, the juxtacanalicular tissue (JCT), as well as the inner layers of the TM plays a key role in the regulation of IOP and there is also a great deal of evidence that there are changes to the ECM of the TM in glaucoma. Increased deposition of ECM proteins in the TM, increased AH outflow resistance, and increased IOP are all associated with POAG (Rohen and Witmer, 1972; Lütjen-Drecoll, 1999). Matrix stiffness is a critical component to a tissue’s function as it can be perceived by cells and cause intracellular responses such as intracellular signals to control gene transcription, protein expression, and cell behavior. The matrix stiffness is also dependent on the type of ECM proteins present as well as the morphology and organization of the ECM itself. The glaucomatous TM has increased deposition of fibronectin and fine fibrillar material (Lütjen-Drecoll et al., 1986; Babizhayev and Brodskaya, 1993; Rohen et al., 1993). This demonstrates that the ECM architecture of the TM is important in regulating aqueous humor outflow and IOP.

## Association of Toll-Like Receptor 4 With Glaucoma

Glaucoma is a complex disease and is well known to have genetic heterogeneity with multiple chromosomal loci linked to the disease. However, the complex molecular mechanisms leading to disease pathology are not fully understood. Here we review the role of toll-like receptor 4 (TLR4) in the development of glaucomatous trabecular meshwork damage. In human glaucomatous donor eyes several toll-like receptors, including TLR4, have been shown to have upregulated expression in the retina after IOP elevation (Luo et al., 2010; Rieck, 2013). Additionally, *TLR4* polymorphisms are indicated to be involved with slower responses to infection, reduced autoimmunity, and glaucoma (Arbour et al., 2000; Radstake et al., 2004). Specifically, association of *TLR4* gene polymorphisms have been identified in Chinese and Japanese cohorts with POAG, normal-tension, and exfoliation glaucoma (Shibuya et al., 2008; Chen et al., 2012; Takano et al., 2012). These data suggest that TLR4 may have a significant role in the cellular pathogenesis of multiple types of glaucoma.

## Toll-Like Receptor 4 Signaling

Toll-like receptors (TLRs) play a significant role in the detection of pathogen associated molecular patterns (PAMPs) and damage associated molecular patterns (DAMPs) (Miller et al., 2015). Functional TLRs 1-10 have been identified in humans and TLRs 1-13 in murine species (Szabo et al., 2006; Yarovinsky, 2014; Nie et al., 2018). Humans have a nonfunctional TLR11 and do not express TLR12 and 13. Unlike TLR3, 7, 8, and 9 which are found in endosomes, TLR4, like TLR1, 2, 5, 6, and 10 are found in the cell membrane. Like most receptors, TLRs require homodimerization or heterodimerization of receptors to activate transcription factors that ultimately lead to the production of chemokines and cytokines. Ultimately, all mammalian TLRs have an extracellular domain that contains leucine-rich domains and an intracellular domain that activates a signaling cascade leading to nuclear factor-kappa beta (NF-κB) activation and translocation to the nucleus (Medzhitov et al., 1997; Chaudhary et al., 1998; Medzhitov et al., 1998; Rock et al., 1998; Chow et al., 1999; Janeway and Medzhitov, 1999; Yang et al., 2000).

TLR4 was first identified as the receptor for lipopolysaccharide (LPS) and was shown to play a vital role in innate immunity (Poltorak et al., 1998). Activation of TLR4 by LPS occurs when the ligand LPS binds to circulating LPS-binding protein (LBP). The LPS bound to LBP binds to TLR4, forming the LPS-TLR4 receptor complex. Once TLR4 is activated, downstream adaptor molecules bind to the Toll/Interleukin-1 receptor (TIR) domain of TLR4. TLR4 requires four adaptor molecules to transduce signals from its TIR domain, with a key adapter being myeloid differentiation factor 88 (MyD88). Signal transduction initiation leads to the activation of NF-κB (Kawai and Akira, 2010). Once activated, NF-κB moves into the nucleus where it initiates both pro-fibrotic and pro-inflammatory gene transcription, such as fibronectin (FN1), interleukin 1 (IL-1), and tumor necrosis factor alpha (TNF-α). TLR4 can also activate the MyD88-independent signaling pathway following endocytosis. This mechanism of endocytosis of LPS bound TLR4 and its degradation through the ubiquitin pathway is one of the negative regulatory mechanisms in the LPS induced TLR4 pathway, and the purpose of this internalization is to limit the receptor expression and the signaling of the pro-inflammatory MyD88 dependent pathway.

**FIGURE 1:**
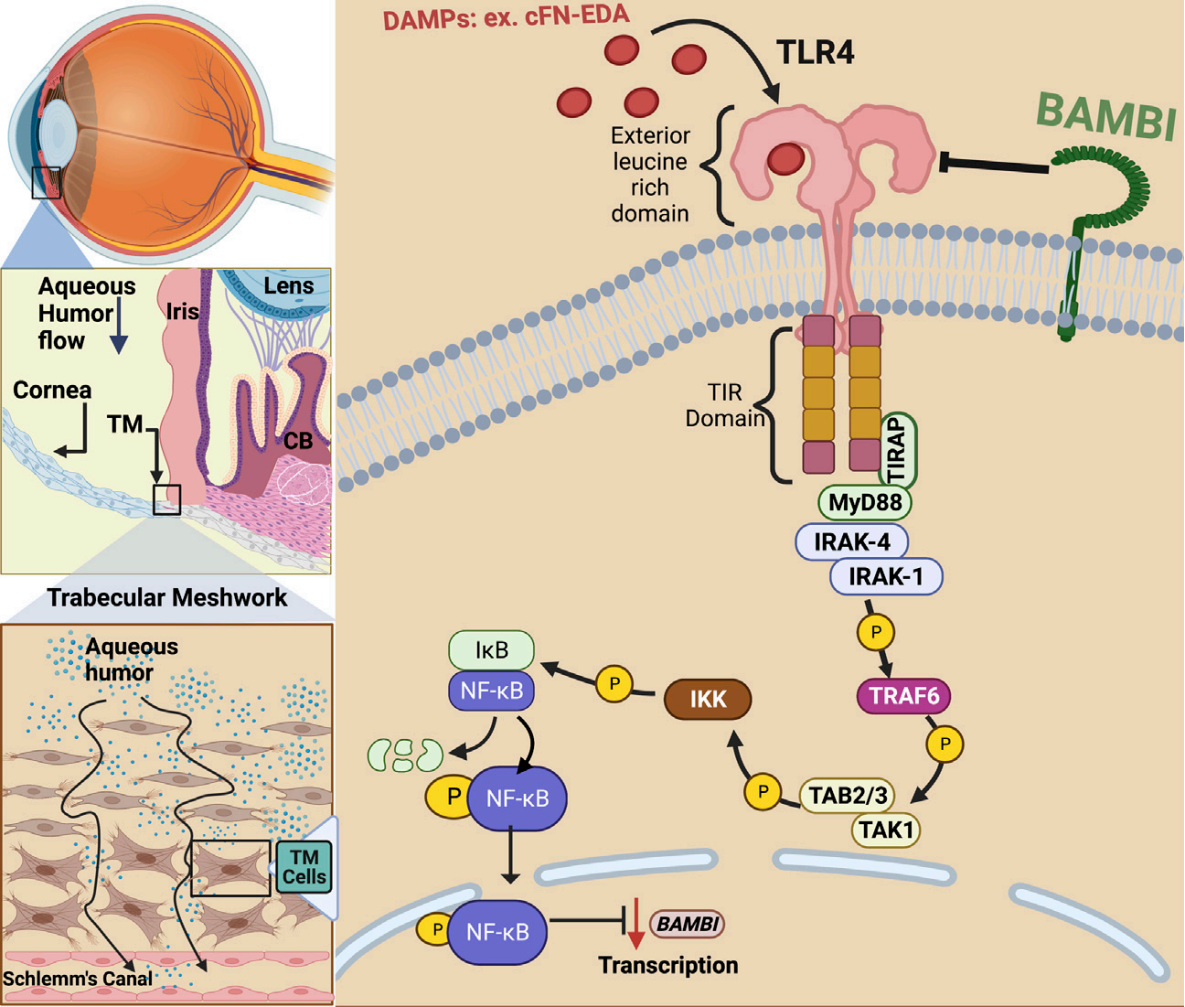
TLR4 activation in the trabecular meshwork. In primary open-angle glaucoma, damage to the TM and inner wall of Schlemm’s canal (SC) endothelium prevents sufficient aqueous humor outflow leading to elevated IOP. Damage associated molecular patterns (DAMPs) such as cellular fibronectin containing EDA isoform (cFN-EDA) are produced from TM tissue damage and excess TGFβ2 signaling and can activate TLR4 leading to Nuclear Factor-Kappa Beta (NF-κB) activation and downregulation of BMP and Activin Membrane Bound Inhibitor (BAMBI) expression.

In addition to the classical activation by exogenous LPS, TLR4 can also be activated by endogenous ligands known as damage-associated molecular patterns (DAMPs) (Figure 1). DAMPs are formed *in situ* because of cell damage, cell injury, and remolding of the ECM (Miyake, 2007; Piccinini and Midwood, 2010). DAMPs can upregulate as well as amplify fibrotic responses in diseases such as renal and hepatic fibrosis, lesional skin and lung in scleroderma patients, as well as in *Tlr4* mutant mice, and augment TGFβ-1 signaling (Poltorak et al., 1998; Seki et al., 2007; Pulskens et al., 2010; Campbell et al., 2011; Bhattacharyya et al., 2013). Endogenous DAMP ligands in the TM can include cellular fibronectin containing the EDA isoform (FN-EDA), high-mobility group box (HMGB)-1, low molecular weight hyaluronic acid (LMWHA), and others (Piccinini and Midwood, 2010; Bhattacharyya et al., 2013). The specific interaction between TLR4 and each of these DAMPs is not completely understood. It is known that HMGB1 binds TLR4 and then signals through adaptor molecules via the Toll/IL-1 receptor-domain to MyD88, IRAK, TRAF and finally to NF-κB (Yang et al., 2010). Specifically, how hyaluronic acid and TLR4 interact is still not known, but it is known that this interaction requires the co-receptors MD2 and CD14 (Jiang et al., 2011). In patients with POAG, high molecular weight hyaluronic acid has been shown to be depleted in the TM; however, the amount of low molecular weight hyaluronic acid in the TM of POAG patients remains to be elucidated (Knepper et al., 1996). An extracellular matrix protein responsible for persistence organ fibrosis known as Tenascin-C directly works with TLR4 and this interaction leads to the activation of NF-κB (Bhattacharyya et al., 2016; Zuliani-Alvarez et al., 2017). Like HMGB-1, FN-EDA also activates TLR4 leading to NF-κB activation. However, whether the signaling pathway is exclusively through the MyD88-dependent pathway is still not known.

Evidence continues to surface that links DAMP activated TLR4 signaling to the regulation and production of ECM proteins in hepatic fibrosis and to TM damage and ocular hypertension (Seki et al., 2007; Bhattacharyya et al., 2013; Hernandez et al., 2017). Regarding fibrosis, specific SNP alleles in TLR4 have been shown to have an overall protective effect and be associated with a delayed progression of fibrosis in liver disease (Huang et al., 2007; Li et al., 2009). As mentioned, DAMPs can activate TLR4 and in doing so, they augment TGFβ signaling and downstream fibrotic responses (Bhattacharyya et al., 2013; Hernandez et al., 2017). DAMPs have also been shown to control the inflammatory and downstream fibrotic response in ischemic wounds when they bind TLR4 (Brancato et al., 2013). TLR4 activation also downregulates the TGFβ pseudoreceptor known as BMP and the activin membrane-bound inhibitor (BAMBI). Bone morphogenic proteins (BMPs) are a group of growth factors that are involved in regulating the ECM and importantly, BMPs can lower ECM deposition caused by TGFβ2 activation (Fuchshofer et al., 2007). BAMBI functions to inhibit TGFβ as well as BMP and activin signaling (Seki et al., 2007; Yan et al., 2009; Bhattacharyya et al., 2013). It is known that BAMBI functions to inhibit TGFβ signaling by cooperating with SMAD7 and impairing SMAD3 activation, while knockdown of *Bambi* expression enhances TGFβ signaling (Yan et al., 2009). In addition, BAMBI can interact directly with either BMP receptors or TGFβ receptors to antagonize downstream signaling (Lin et al., 2006). We have shown that when *Bambi* is conditionally knocked down in the TM, IOP becomes elevated in mice (Hernandez et al., 2018). Downregulation of *Bambi* by TLR4 is controlled by the NFκB-dependent signaling pathway (Seki et al., 2007; Guo and Friedman, 2010; Yang and Seki, 2012). Activation of TLR4 therefore downregulates *Bambi* resulting in unopposed TGFβ signaling and fibrogenesis. This leads to an upregulation and subsequent accumulation of DAMPs, creating a feed-forward loop and further amplification and continuation of the fibrotic response via TGFβ signaling.

## Crosstalk of TGFβ2–TLR4 Signaling

**FIGURE 2:**
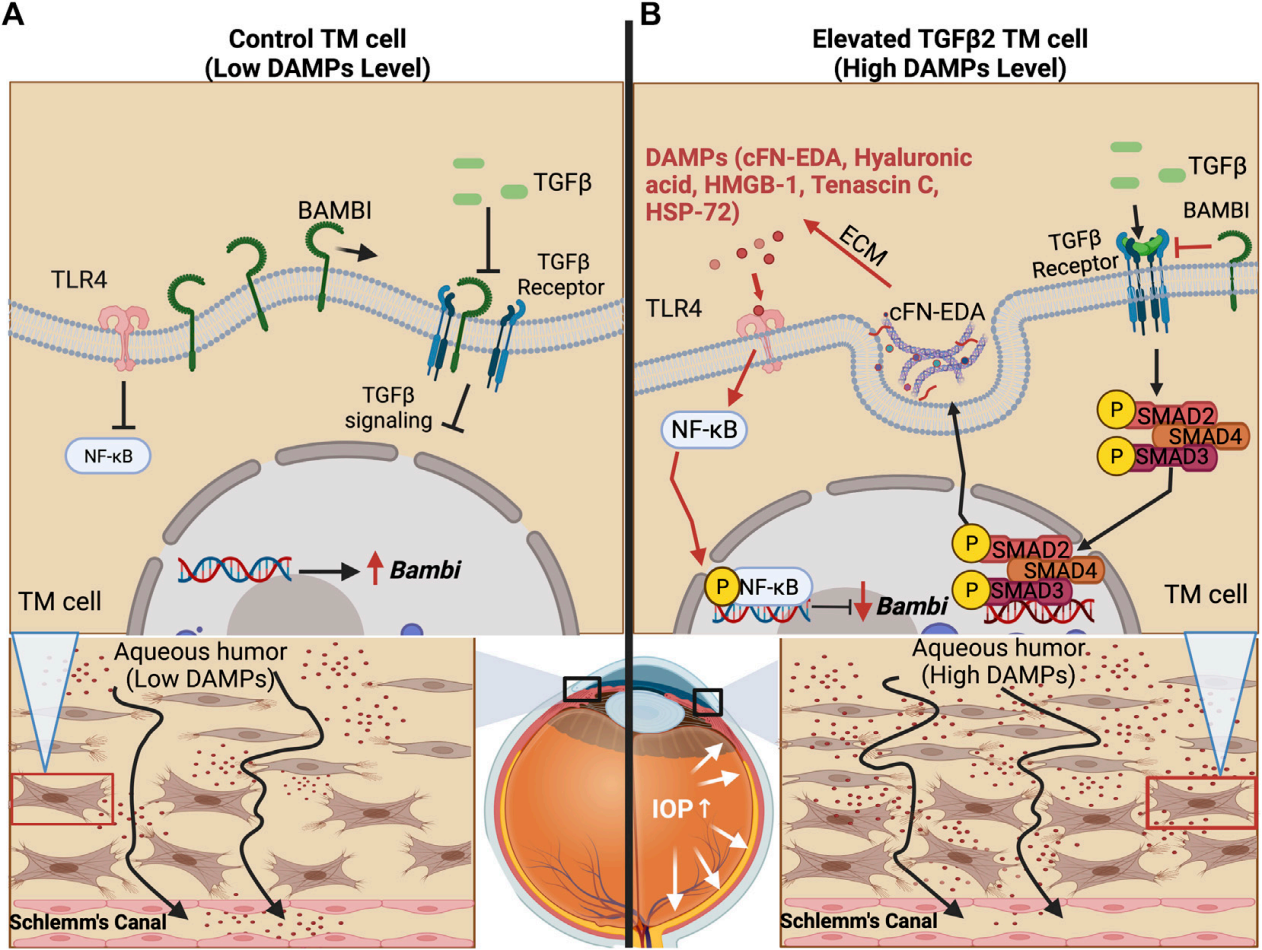
TLR4 and TGFβ2 signaling crosstalk. **(A)** The trabecular meshwork under homeostasis expresses basal levels of extracellular matrix proteins and BMP and Activin Membrane Bound Inhibitor (BAMBI), which inhibits endogenous TGFβ2 signaling. **(B)** Bioactivated TGFβ2 leads to the expression of DAMPs, including cFN-EDA and LMW Hyaluronic acid, which activate TLR4 and lead to subsequent NF-κB activation. Phosphorylated NF-κB lowers the expression of BAMBI, which in turn, leads to uninhibited TGFβ2 signaling.

TGFβ2 is a profibrotic cytokine that when in its bioactivated form upregulates ECM proteins, some of which are FN, elastin, and several forms of collagens. In a healthy eye, TGFβ2 is the predominant isoform of TGFβ. TGFβ is known to induce various growth factors, such as connective tissue growth factor (CTGF) and fibroblast growth factors (FGFs) (Saika, 2006) and helps maintain tissue homeostasis in the TM regulating ECM synthesis, deposition, and degradation (Sethi et al., 2011b). Importantly, these factors have roles in restoration of normal tissue following injury. In addition to its ability to influence ECM remodeling, TGFβ is also known to affect multiple cellular processes, from cell growth to apoptosis (Chen and Ten Dijke, 2016). However, uninhibited and increased TGFβ2 signaling can lead to deleterious effects. Elevated TGFβ2 signaling results in damage to the ECM of the TM and increased stiffness of the TM (Russell and Johnson, 2012; Vranka et al., 2018). TGFβ2 is highly elevated in the aqueous humor of glaucoma patients and plays a vital role in the development of POAG (Tripathi et al., 1994; Inatani et al., 2001; Ochiai and Ochiai, 2002; Ozcan et al., 2004). We and others have shown that treatment of TM cells with TGFβ2 induces cross-linking of the ECM as well as alteration in the composition of the ECM (Welge-Lüssen et al., 1999; Fleenor et al., 2006; Fuchshofer et al., 2007; Wordinger et al., 2007; Sethi et al., 2011a; Tovar-Vidales et al., 2011; Hernandez et al., 2017). In anterior segment perfusion organ culture models the addition of TGFβ2 elevates IOP and overexpression of TGFβ2 in mouse eyes causes ocular hypertension (Gottanka et al., 2004; Shepard et al., 2010; Hernandez et al., 2017). In human TM cells, TGFβ2 signals through the canonical SMAD and non-SMAD pathways and also alters the ECM (Sethi et al., 2011a; Tovar-Vidales et al., 2011; Zode et al., 2011). Specifically, TGFβ2 causes phosphorylation of a SMAD signaling complex. This complex then moves into the nucleus, leading to the induction of pro-fibrotic gene transcription, which causes an increase in the production of fibrotic factors, such as the various ECM components in the TM. For ocular hypertension to occur in mice, TGFβ2 signaling through the canonical SMAD pathway is essential (McDowell et al., 2013). Taken together, this indicates that the effects of TGFβ2 signaling are a major component in the development of ocular hypertension and that TGFβ2 regulates the expression of ECM proteins in the TM. Our group has shown that there is crosstalk between the TGFβ2 and TLR4 signaling pathways in the TM and that this crosstalk is contributing to glaucomatous ocular hypertension (Figure 2) (Hernandez et al., 2017). Although studies in the TM have focused primarily on the canonical SMAD-dependent TGFβ2 signaling pathway in the context of TGFβ2-TLR4 signaling crosstalk, work in other tissues has indicated that TLR4 activation can also effect non-canonical TGFβ pathways as well (McKeown-Longo and Higgins, 2017). However, whether changes in the TGFβ2 pathway occur first to induce crosstalk or if TLR4 induction first occurs to facilitate crosstalk between the pathways, is still not completely clear. It is known that increased TGFβ2 levels in glaucoma may be due to epigenetics (Bermudez et al., 2016), suggesting that it is the increased TGFβ signaling that occurs first leading to production of excess ECM and DAMPs, which would then activate TLR4 leading to a feedforward signaling loop.

As mentioned, TGFβ2 signaling increases the production of ECM proteins, including FN. We and others have identified FN, a dimeric multidomain ECM glycoprotein, to be elevated in glaucomatous TM tissues and aqueous humor (Faralli et al., 2009; Hernandez et al., 2017). Fibronectin functions as a regulator of cellular processes, directs and maintains tissue organization and ECM composition, directs ECM-ECM and ECM-cell interactions, and regulates activity of growth factors and proteins associated with ECM remodeling. The multi-domain dimer is composed of type I, type II, and type III domains with over twenty alternatively spliced isoforms. FN is composed of either cellular FN or plasma FN isoforms. Cellular FN has multiple isoforms generated by alternative processing of a single primary transcript at three domains: extra domain A (EDA), extra domain B (EDB), and the type III homologies connecting segment (White et al., 2008). The expression of FN-EDA is upregulated as a response to tissue injury, repair, or remodeling, and during disease states (Kuhn et al., 1989; Muro et al., 2003). The FN-EDA isoform is elevated in glaucomatous TM tissue compared to normal TM tissue and amplifies the response of TGFβ2 in primary TM cells in culture (Medina-Ortiz et al., 2013; Hernandez et al., 2017). Importantly, FN-EDA acts as an endogenous ligand (DAMP) for TLR4 (Okamura et al., 2001). We have identified FN-EDA as an important regulator of pathogenic TLR4 and TGFβ2 signaling in the TM (Hernandez et al., 2017; Roberts et al., 2020). Importantly, the consequence of the continuous activation of TLR4 due to this endogenous ligand is the subsequent uninhibited TGFβ2 signaling and an amplification of the fibrotic response in the TM. The activation of TLR4 is known to be dependent on the expression of MD-2 and other TLR4 accessory proteins (Yang et al., 2000; Okamura et al., 2001). The α4β1 integrin has been identified to function as a TLR4-coreceptor to initiate a FN-EDA dependent response in fibroblasts and it is known that FN-EDA contains integrin α4β1 binding sites (Liao et al., 2002; Kelsh-Lasher et al., 2017). Studies on pathogen-initiated TLR4 signaling suggest that adhesion receptors may play important roles in the regulation of the TLR4-mediated fibrotic response to tissue damage, so this may be a route that FN-EDA utilizes to elicit a TLR4 mediated response in TM cells (Gianni et al., 2012; Ling et al., 2014; Casiraghi et al., 2016).

## TLR4 Signaling in the TM

Recently, utilizing a selective inhibitor of TLR4 signaling, TAK-242, we showed TGFβ2 induced ECM production in the TM was inhibited (Hernandez et al., 2017). Notably, FN-EDA amplified TGFβ2 ECM deposition and TAK-242 blocked this effect. To evaluate the role of FN-EDA in the development of ocular hypertension, we utilized an adenovirus vector to overexpress bioactivated TGFβ2 in the TM of mice containing a constitutively active FN-EDA isoform or in FN-EDA null mice, with or without mutation in *Tlr4*. Here we found that TGFβ2-induced ocular hypertension and ECM production is dependent on both EDA and Tlr4, and in mice constitutively expressing FN-EDA the effects of TGFβ2 are amplified (Roberts et al., 2020). To further evaluate the link between the TGFβ2–TLR4 pathway in the TM, we focused our attention on the role of Bambi, which is known to be downregulated via NF-kB signaling after TLR4 activation. We demonstrated that conditional knock-out of *Bambi* in the TM resulted in increased ECM deposition and development of ocular hypertension, likely due to uninhibited TGFβ2 signaling. In addition, we also tested the role of NF-κB, an upstream regulator of *Bambi* expression, in TGFβ2-induced ocular hypertension and found that mutation in the p50 subunit of NF-κB prevented TGFβ2-induced ocular hypertension (Hernandez et al., 2020). These data suggest that TGFβ2-TLR4 signaling crosstalk is important in the development of ocular hypertension and ECM changes in the TM.

We have also examined ways to attenuate the downstream fibrotic signaling initiated by TGFβ2–TLR4 signaling crosstalk. Since NF-κB is necessary for this fibrotic response, we examined how a suppressor of NF-κB signaling, A20, may be able to rescue the effects of TGFβ2 and TLR4 activators, such as FN-EDA, within the TM. Previous work showed that A20 is downregulated in human TM cells that expressed constitutively active α5β3 integrin (Filla et al., 2021). The activation of this integrin is suggested to contribute to the fibrotic-like changes observed in POAG, therefore A20’s diminished presence may be contributing to the increased fibrotic response seen in the glaucomatous TM. Expression changes in A20 have also been shown in the retina of glaucomatous human donor eyes (Yang et al., 2011). We showed that TGFβ2 causes a decrease in the expression of A20 in TM cells, while at the same time increasing expression of ECM proteins such as FN (Mzyk et al., 2022). Overexpression of A20 in human TM cells attenuated the amount of FN expressed in TM cells after stimulation with either TGFβ2, LPS, or FN-EDA (Mzyk et al., 2022). These data suggest that A20 is a novel molecular target that inhibits the pathological ECM changes in the glaucomatous TM.

## Conclusion

This review summarizes the involvement of the TGFβ2-TLR4 signaling pathways in augmenting the pathogenesis of ocular hypertension at the trabecular meshwork. The TLR4 pathway is a fibroinflammatory pathway that can modulate the function of the TM, specifically by altering the TM’s rate of deposition of ECM, leading to the impairment of aqueous humor outflow and the progression of glaucoma. Identification of additional DAMPs and regulators of TLR4 signaling may allow us to identify potential therapeutic targets for POAG. Further investigation of TGFβ2-TLR4 crosstalk in the TM will help to explain the mechanisms involved in the development of glaucomatous TM damage.

## References

[B1] Arbour N. C. Lorenz E. Schutte B. C. Zabner J. Kline J. N. Jones M. (2000). TLR4 Mutations Are Associated with Endotoxin Hyporesponsiveness in Humans. Nat. Genet. 25, 187–191. 10.1038/76048 PubMed Abstract | 10.1038/76048 | Google Scholar 10835634

[B2] Babizhayev M. A. Brodskaya M. W. (1993). Immunohistochemical Monitoring of the Effect of a Synthetic Fibronectin-like Peptide (ArgGlyAsp) on the Age-Related Changes in the Isolated Human Corneoscleral Tissue of Glaucomatous Eyes. Mech. Ageing Dev. 72, 1–12. 10.1016/0047-6374(93)90126-c PubMed Abstract | 10.1016/0047-6374(93)90126-c | Google Scholar 7509429

[B3] Bermudez J. Y. Webber H. C. Patel G. C. Liu X. Cheng Y.-Q. Clark A. F. (2016). HDAC Inhibitor-Mediated Epigenetic Regulation of Glaucoma-Associated TGFβ2 in the Trabecular Meshwork. Invest. Ophthalmol. Vis. Sci. 57, 3698–3707. 10.1167/iovs16-19446 PubMed Abstract | 10.1167/iovs16-19446 | Google Scholar 27403998 PMC4973502

[B4] Bhattacharyya S. Kelley K. Melichian D. S. Tamaki Z. Fang F. Su Y. (2013). Toll-Like Receptor 4 Signaling Augments Transforming Growth Factor-β Responses. Am. J. Pathology 182, 192–205. 10.1016/j.ajpath.2012.09.007 10.1016/j.ajpath.2012.09.007 | Google Scholar PMC3538029 23141927

[B5] Bhattacharyya S. Wang W. Morales-Nebreda L. Feng G. Wu M. Zhou X. (2016). Tenascin-C Drives Persistence of Organ Fibrosis. Nat. Commun. 7, 11703. 10.1038/ncomms11703 PubMed Abstract | 10.1038/ncomms11703 | Google Scholar 27256716 PMC4895803

[B6] Brancato S. K. Thomay A. A. Daley J. M. Crane M. J. Reichner J. S. Sabo E. (2013). Toll-like Receptor 4 Signaling Regulates the Acute Local Inflammatory Response to Injury and the Fibrosis/neovascularization of Sterile Wounds. Wound Repair Regen. 21, 624–633. 10.1111/wrr.12061 PubMed Abstract | 10.1111/wrr.12061 | Google Scholar 23758142 PMC4469904

[B7] Campbell M. T. Hile K. L. Zhang H. Asanuma H. Vanderbrink B. A. Rink R. R. (2011). Toll-like Receptor 4: a Novel Signaling Pathway during Renal Fibrogenesis. J. Surg. Res. 168, e61–e69. 10.1016/j.jss.2009.09.053 PubMed Abstract | 10.1016/j.jss.2009.09.053 | Google Scholar 20089260 PMC2888706

[B8] Casiraghi C. Gianni T. Campadelli-Fiume G. (2016). αvβ3 Integrin Boosts the Innate Immune Response Elicited in Epithelial Cells through Plasma Membrane and Endosomal Toll-like Receptors. J. Virol. 90, 4243–4248. 10.1128/jvi.03175-15 PubMed Abstract | 10.1128/jvi.03175-15 | Google Scholar 26842473 PMC4810540

[B9] Chaudhary P. M. Ferguson C. Nguyen V. Nguyen O. Massa H. F. Eby M. (1998). Cloning and Characterization of Two Toll/Interleukin-1 Receptor-like Genes TIL3 and TIL4: Evidence for a Multi-Gene Receptor Family in Humans. Blood 91, 4020–4027. 10.1182/blood.v91.11.4020.411a44_4020_4027 PubMed Abstract | 10.1182/blood.v91.11.4020.411a44_4020_4027 | Google Scholar 9596645

[B10] Chen Lj Tam Po Leung Dy Fan Ah Zhang M. Tham Cc (2012). SNP Rs1533428 at 2p16.3 as a Marker for Late-Onset Primary Open-Angle Glaucoma. Mol. Vis. 18, 1629–1639. PubMed Abstract | Google Scholar 22773901 PMC3388985

[B11] Chen W. Ten Dijke P. (2016). Immunoregulation by Members of the TGFβ Superfamily. Nat. Rev. Immunol. 16, 723–740. 10.1038/nri.2016.112 PubMed Abstract | 10.1038/nri.2016.112 | Google Scholar 27885276

[B12] Chow J. C. Young D. W. Golenbock D. T. Christ W. J. Gusovsky F. (1999). Toll-like Receptor-4 Mediates Lipopolysaccharide-Induced Signal Transduction. J. Biol. Chem. 274, 10689–10692. 10.1074/jbc.274.16.10689 PubMed Abstract | 10.1074/jbc.274.16.10689 | Google Scholar 10196138

[B13] Faralli J. A. Schwinn M. K. Gonzalez J. M. Jr. Filla M. S. Peters D. M. (2009). Functional Properties of Fibronectin in the Trabecular Meshwork. Exp. Eye Res. 88, 689–693. 10.1016/j.exer.2008.08.019 PubMed Abstract | 10.1016/j.exer.2008.08.019 | Google Scholar 18835267 PMC2693904

[B14] Filla M. S. Meyer K. K. Faralli J. A. Peters D. M. (2021). Overexpression and Activation of Alphavbeta3 Integrin Differentially Affects TGFbeta2 Signaling in Human Trabecular Meshwork Cells. Cells 10, 1923. 10.3390/cells10081923 PubMed Abstract | 10.3390/cells10081923 | Google Scholar 34440692 PMC8394542

[B15] Fleenor D. L. Shepard A. R. Hellberg P. E. Jacobson N. Pang I.-H. Clark A. F. (2006). TGFβ2-Induced Changes in Human Trabecular Meshwork: Implications for Intraocular Pressure. Invest. Ophthalmol. Vis. Sci. 47, 226–234. 10.1167/iovs.05-1060 PubMed Abstract | 10.1167/iovs.05-1060 | Google Scholar 16384967

[B16] Friedman Ds Wolfs Rc O'colmain Bj Klein Be Taylor Hr West S. (2004). Prevalence of Open-Angle Glaucoma Among Adults in the United States. Arch. Ophthalmol. 122, 532–538. 10.1001/archopht.122.4.532 PubMed Abstract | 10.1001/archopht.122.4.532 | Google Scholar 15078671 PMC2798086

[B17] Fuchshofer R. Yu A. H. L. Welge-Lu¨Ssen U. Tamm E. R. (2007). Bone Morphogenetic Protein-7 Is an Antagonist of Transforming Growth Factor-Β2 in Human Trabecular Meshwork Cells. Invest. Ophthalmol. Vis. Sci. 48, 715–726. 10.1167/iovs.06-0226 PubMed Abstract | 10.1167/iovs.06-0226 | Google Scholar 17251470

[B18] Gianni T. Leoni V. Chesnokova L. S. Hutt-Fletcher L. M. Campadelli-Fiume G. (2012). αvβ3-integrin Is a Major Sensor and Activator of Innate Immunity to Herpes Simplex Virus-1. Proc. Natl. Acad. Sci. U.S.A. 109, 19792–19797. 10.1073/pnas.1212597109 PubMed Abstract | 10.1073/pnas.1212597109 | Google Scholar 23150579 PMC3511702

[B19] Gong H. Tripathi R. C. Tripathi B. J. (1996). Morphology of the Aqueous Outflow Pathway. Microsc. Res. Tech. 33, 336–367. 10.1002/(sici)1097-0029(19960301)33:4<336::aid-jemt4>3.0.co;2-n PubMed Abstract | 10.1002/(sici)1097-0029(19960301)33:4<336::aid-jemt4>3.0.co;2-n | Google Scholar 8652890

[B20] Gottanka J. Chan D. Eichhorn M. Lutjen-Drecoll E. Ethier C. R. (2004). Effects of TGF- 2 in Perfused Human Eyes. Investigative Ophthalmol. Vis. Sci. 45, 153–158. 10.1167/iovs.03-0796 PubMed Abstract | 10.1167/iovs.03-0796 | Google Scholar 14691167

[B21] Guo J. Friedman S. L. (2010). Toll-like Receptor 4 Signaling in Liver Injury and Hepatic Fibrogenesis. Fibrogenes. Tissue Repair 3, 21. 10.1186/1755-1536-3-21 PubMed Abstract | 10.1186/1755-1536-3-21 | Google Scholar PMC2984459 20964825

[B22] Hernandez H. Medina-Ortiz W. E. Luan T. Clark A. F. Mcdowell C. M. (2017). Crosstalk between Transforming Growth Factor Beta-2 and Toll-like Receptor 4 in the Trabecular Meshwork. Invest. Ophthalmol. Vis. Sci. 58, 1811–1823. 10.1167/iovs.16-21331 PubMed Abstract | 10.1167/iovs.16-21331 | Google Scholar 28346614 PMC5374883

[B23] Hernandez H. Millar J. C. Curry S. M. Clark A. F. Mcdowell C. M. (2018). BMP and Activin Membrane Bound Inhibitor Regulates the Extracellular Matrix in the Trabecular Meshwork. Invest. Ophthalmol. Vis. Sci. 59, 2154–2166. 10.1167/iovs.17-23282 PubMed Abstract | 10.1167/iovs.17-23282 | Google Scholar 29801150 PMC5915111

[B24] Hernandez H. Roberts A. L. Mcdowell C. M. (2020). Nuclear Factor-Kappa Beta Signaling Is Required for Transforming Growth Factor Beta-2 Induced Ocular Hypertension. Exp. Eye Res. 191, 107920. 10.1016/j.exer.2020.107920 PubMed Abstract | 10.1016/j.exer.2020.107920 | Google Scholar 31923415 PMC7015797

[B25] Hogan M. J. Alvarado J. Weddell J. E. (1971). Histology of the Human Ey: An Atlas and Textbook. Saunders: The University of Michigan. Google Scholar

[B26] Huang H. Shiffman M. L. Friedman S. Venkatesh R. Bzowej N. Abar O. T. (2007). A 7 Gene Signature Identifies the Risk of Developing Cirrhosis in Patients with Chronic Hepatitis C. Hepatology 46, 297–306. 10.1002/hep.21695 PubMed Abstract | 10.1002/hep.21695 | Google Scholar 17461418

[B27] Inatani M. Tanihara H. Katsuta H. Honjo M. Kido N. Honda Y. (2001). Transforming Growth Factor-Β2 Levels in Aqueous Humor of Glaucomatous Eyes. Graefe's Arch. Clin. Exp. Ophthalmol. 239, 109–113. 10.1007/s004170000241 10.1007/s004170000241 | Google Scholar 11372538

[B28] Janeway C. A. Jr. Medzhitov R. (1999). Innate Immunity: Lipoproteins Take Their Toll on the Host. Curr. Biol. 9, R879–R882. 10.1016/s0960-9822(00)80073-1 PubMed Abstract | 10.1016/s0960-9822(00)80073-1 | Google Scholar 10607553

[B29] Jiang D. Liang J. Noble P. W. (2011). Hyaluronan as an Immune Regulator in Human Diseases. Physiol. Rev. 91, 221–264. 10.1152/physrev.00052.2009 PubMed Abstract | 10.1152/physrev.00052.2009 | Google Scholar 21248167 PMC3051404

[B30] Kawai T. Akira S. (2010). The Role of Pattern-Recognition Receptors in Innate Immunity: Update on Toll-like Receptors. Nat. Immunol. 11, 373–384. 10.1038/ni.1863 PubMed Abstract | 10.1038/ni.1863 | Google Scholar 20404851

[B31] Keller K. E. Aga M. Bradley J. M. Kelley M. J. Acott T. S. (2009). Extracellular Matrix Turnover and Outflow Resistance. Exp. Eye Res. 88, 676–682. 10.1016/j.exer.2008.11.023 PubMed Abstract | 10.1016/j.exer.2008.11.023 | Google Scholar 19087875 PMC2700052

[B32] Kelsh-Lasher R. M. Ambesi A. Bertram C. Mckeown-Longo P. J. (2017). Integrin α4β1 and TLR4 Cooperate to Induce Fibrotic Gene Expression in Response to Fibronectin's EDA Domain. J. Investigative Dermatology 137, 2505–2512. 10.1016/j.jid.2017.08.005 PubMed Abstract | 10.1016/j.jid.2017.08.005 | Google Scholar PMC6736604 28842322

[B33] Kingman S. (2004). Glaucoma Is Second Leading Cause of Blindness Globally. Bull. World Health Organ 82, 887–888. 10.1590/S0042-96862004001100019 PubMed Abstract | 10.1590/S0042-96862004001100019 | Google Scholar 15640929 PMC2623060

[B34] Knepper Pa Goossens W. Hvizd M. Palmberg P. F. (1996). Glycosaminoglycans of the Human Trabecular Meshwork in Primary Open-Angle Glaucoma. Invest. Ophthalmol. Vis. Sci. 37, 1360–1367. PubMed Abstract | Google Scholar 8641839

[B35] Kuhn C. Boldt J. King T. E. Jr. Crouch E. Vartio T. Mcdonald J. A. (1989). An Immunohistochemical Study of Architectural Remodeling and Connective Tissue Synthesis in Pulmonary Fibrosis. Am. Rev. Respir. Dis. 140, 1693–1703. 10.1164/ajrccm/140.6.1693 PubMed Abstract | 10.1164/ajrccm/140.6.1693 | Google Scholar 2604297

[B36] Li Y. Chang M. Abar O. Garcia V. Rowland C. Catanese J. (2009). Multiple Variants in Toll-like Receptor 4 Gene Modulate Risk of Liver Fibrosis in Caucasians with Chronic Hepatitis C Infection. J. Hepatology 51, 750–757. 10.1016/j.jhep.2009.04.027 PubMed Abstract | 10.1016/j.jhep.2009.04.027 | Google Scholar PMC2883297 19586676

[B37] Liao Y.-F. Gotwals P. J. Koteliansky V. E. Sheppard D. Van De Water L. (2002). The EIIIA Segment of Fibronectin Is a Ligand for Integrins α9β1 and α4β1Providing a Novel Mechanism for Regulating Cell Adhesion by Alternative Splicing. J. Biol. Chem. 277, 14467–14474. 10.1074/jbc.m201100200 PubMed Abstract | 10.1074/jbc.m201100200 | Google Scholar 11839764

[B38] Lin S. J. Lerch T. F. Cook R. W. Jardetzky T. S. Woodruff T. K. (2006). The Structural Basis of TGF-β, Bone Morphogenetic Protein, and Activin Ligand Binding. Reproduction 132, 179–190. 10.1530/rep.1.01072 PubMed Abstract | 10.1530/rep.1.01072 | Google Scholar 16885528

[B39] Ling G. S. Bennett J. Woollard K. J. Szajna M. Fossati-Jimack L. Taylor P. R. (2014). Integrin CD11b Positively Regulates TLR4-Induced Signalling Pathways in Dendritic Cells but Not in Macrophages. Nat. Commun. 5, 3039. 10.1038/ncomms4039 PubMed Abstract | 10.1038/ncomms4039 | Google Scholar 24423728 PMC3905776

[B40] Lu Lj Tsai Jc Liu J. (2017). Novel Pharmacologic Candidates for Treatment of Primary Open-Angle Glaucoma. Yale J. Biol. Med. 90, 111–118. PubMed Abstract | Google Scholar 28356898 PMC5369028

[B41] Luo C. Yang X. Kain A. D. Powell D. W. Kuehn M. H. Tezel G. (2010). Glaucomatous Tissue Stress and the Regulation of Immune Response through Glial Toll-like Receptor Signaling. Invest. Ophthalmol. Vis. Sci. 51, 5697–5707. 10.1167/iovs.10-5407 PubMed Abstract | 10.1167/iovs.10-5407 | Google Scholar 20538986 PMC3061506

[B42] Lütjen-Drecoll E. (1999). Functional Morphology of the Trabecular Meshwork in Primate Eyes. Prog. Retin. Eye Res. 18, 91–119. 10.1016/s1350-9462(98)00011-1 PubMed Abstract | 10.1016/s1350-9462(98)00011-1 | Google Scholar 9920500

[B43] Lütjen-Drecoll E. Shimizu T. Rohrbach M. Rohen J. W. (1986). Quantitative Analysis of 'plaque Material' in the Inner- and Outer Wall of Schlemm's Canal in Normal- and Glaucomatous Eyes. Exp. Eye Res. 42, 443–455. 10.1016/0014-4835(86)90004-7 PubMed Abstract | 10.1016/0014-4835(86)90004-7 | Google Scholar 3720863

[B44] McDowell C. M. Tebow H. E. Wordinger R. J. Clark A. F. (2013). Smad3 Is Necessary for Transforming Growth Factor-Beta2 Induced Ocular Hypertension in Mice. Exp. Eye Res. 116, 419–423. 10.1016/j.exer.2013.10.017 PubMed Abstract | 10.1016/j.exer.2013.10.017 | Google Scholar 24184030 PMC3895953

[B45] McKeown-Longo P. J. Higgins P. J. (2017). Integration of Canonical and Noncanonical Pathways in TLR4 Signaling: Complex Regulation of the Wound Repair Program. Adv. Wound Care 6, 320–329. 10.1089/wound.2017.0736 PubMed Abstract | 10.1089/wound.2017.0736 | Google Scholar PMC5649420 29062589

[B46] Medina-Ortiz W. E. Belmares R. Neubauer S. Wordinger R. J. Clark A. F. (2013). Cellular Fibronectin Expression in Human Trabecular Meshwork and Induction by Transforming Growth Factor-Β2. Invest. Ophthalmol. Vis. Sci. 54, 6779–6788. 10.1167/iovs.13-12298 PubMed Abstract | 10.1167/iovs.13-12298 | Google Scholar 24030464 PMC3799562

[B47] Medzhitov R. Preston-Hurlburt P. Janeway C. A. Jr. (1997). A Human Homologue of the *Drosophila* Toll Protein Signals Activation of Adaptive Immunity. Nature 388, 394–397. 10.1038/41131 PubMed Abstract | 10.1038/41131 | Google Scholar 9237759

[B48] Medzhitov R. Preston-Hurlburt P. Kopp E. Stadlen A. Chen C. Ghosh S. (1998). MyD88 Is an Adaptor Protein in the hToll/IL-1 Receptor Family Signaling Pathways. Mol. Cell 2, 253–258. 10.1016/s1097-2765(00)80136-7 PubMed Abstract | 10.1016/s1097-2765(00)80136-7 | Google Scholar 9734363

[B49] Miller R. E. Belmadani A. Ishihara S. Tran P. B. Ren D. Miller R. J. (2015). Damage-associated Molecular Patterns Generated in Osteoarthritis Directly Excite Murine Nociceptive Neurons through Toll-like Receptor 4. Arthritis & Rheumatology 67, 2933–2943. 10.1002/art.39291 10.1002/art.39291 | Google Scholar 26245312 PMC4626273

[B50] Miyake K. (2007). Innate Immune Sensing of Pathogens and Danger Signals by Cell Surface Toll-like Receptors. Seminars Immunol. 19, 3–10. 10.1016/j.smim.2006.12.002 10.1016/j.smim.2006.12.002 | Google Scholar 17275324

[B51] Morrison J. C. Acott T. S. (2003). “Anatomy and Physiology of Aqueous Humor Outflow,” in Glaucoma: Science and Practice. Editor Gumpert E. (Thieme Medical Publishers). Google Scholar

[B52] Muro A. F. Chauhan A. K. Gajovic S. Iaconcig A. Porro F. Stanta G. (2003). Regulated Splicing of the Fibronectin EDA Exon Is Essential for Proper Skin Wound Healing and Normal Lifespan. J. Cell Biol. 162, 149–160. 10.1083/jcb.200212079 PubMed Abstract | 10.1083/jcb.200212079 | Google Scholar 12847088 PMC2172721

[B53] Mzyk P. Zalog Eg Mcdowell C. M. (2022). A20 Attenuates the Fibrotic Response in the Trabecular Meshwork. Int. J. Mol. Sci. 23. 10.3390/ijms23041928 10.3390/ijms23041928 | Google Scholar PMC8875798 35216043

[B54] Nie L. Cai S.-Y. Shao J.-Z. Chen J. (2018). Toll-Like Receptors, Associated Biological Roles, and Signaling Networks in Non-mammals. Front. Immunol. 9, 1523. 10.3389/fimmu.2018.01523 PubMed Abstract | 10.3389/fimmu.2018.01523 | Google Scholar 30034391 PMC6043800

[B55] Ochiai Y. Ochiai H. (2002). Higher Concentration of Transforming Growth Factor-β in Aqueous Humor of Glaucomatous Eyes and Diabetic Eyes. Jpn. J. Ophthalmol. 46, 249–253. 10.1016/s0021-5155(01)00523-8 PubMed Abstract | 10.1016/s0021-5155(01)00523-8 | Google Scholar 12063033

[B56] Okamura Y. Watari M. Jerud E. S. Young D. W. Ishizaka S. T. Rose J. (2001). The Extra Domain A of Fibronectin Activates Toll-like Receptor 4. J. Biol. Chem. 276, 10229–10233. 10.1074/jbc.m100099200 PubMed Abstract | 10.1074/jbc.m100099200 | Google Scholar 11150311

[B57] Ozcan A. A. Ozdemir N. Canataroglu A. (2004). The Aqueous Levels of TGF- 2 in Patients with Glaucoma. Int. Ophthalmol. 25, 19–22. 10.1023/b:inte.0000018524.48581.79 PubMed Abstract | 10.1023/b:inte.0000018524.48581.79 | Google Scholar 15085971

[B58] Piccinini A. M. Midwood K. S. (2010). DAMPening Inflammation by Modulating TLR Signalling. Mediat. Inflamm. 2010, 672395. 10.1155/2010/672395 PubMed Abstract | 10.1155/2010/672395 | Google Scholar PMC2913853 20706656

[B59] Poltorak A. He X. Smirnova I. Liu M.-Y. Huffel C. V. Du X. (1998). Defective LPS Signaling in C3H/HeJ and C57BL/10ScCr Mice: Mutations in Tlr4 Gene. Science 282, 2085–2088. 10.1126/science.282.5396.2085 PubMed Abstract | 10.1126/science.282.5396.2085 | Google Scholar 9851930

[B60] Pulskens W. P. Rampanelli E. Teske G. J. Butter L. M. Claessen N. Luirink I. K. (2010). TLR4 Promotes Fibrosis but Attenuates Tubular Damage in Progressive Renal Injury. J. Am. Soc. Nephrol. 21, 1299–1308. 10.1681/asn.2009070722 PubMed Abstract | 10.1681/asn.2009070722 | Google Scholar 20595685 PMC2938595

[B61] Quigley H. A. Broman A. T. (2006). The Number of People with Glaucoma Worldwide in 2010 and 2020. Br. J. Ophthalmol. 90, 262–267. 10.1136/bjo.2005.081224 PubMed Abstract | 10.1136/bjo.2005.081224 | Google Scholar 16488940 PMC1856963

[B62] Radstake T. R. D. J. Franke B. Hanssen S. Netea M. G. Welsing P. Barrera P. (2004). The Toll-like Receptor 4 Asp299Gly Functional Variant Is Associated with Decreased Rheumatoid Arthritis Disease Susceptibility but Does Not Influence Disease Severity And/or Outcome. Arthritis & Rheumatism 50, 999–1001. 10.1002/art.20114 10.1002/art.20114 | Google Scholar 15022344

[B63] Rieck J. (2013). The Pathogenesis of Glaucoma in the Interplay with the Immune System. Invest. Ophthalmol. Vis. Sci. 54, 2393–2409. 10.1167/iovs.12-9781 PubMed Abstract | 10.1167/iovs.12-9781 | Google Scholar 23539162

[B64] Roberts A. L. Mavlyutov T. A. Perlmutter T. E. Curry S. M. Harris S. L. Chauhan A. K. (2020). Fibronectin Extra Domain A (FN-EDA) Elevates Intraocular Pressure through Toll-like Receptor 4 Signaling. Sci. Rep. 10, 9815. 10.1038/s41598-020-66756-6 PubMed Abstract | 10.1038/s41598-020-66756-6 | Google Scholar 32555351 PMC7299944

[B65] Rock F. L. Hardiman G. Timans J. C. Kastelein R. A. Bazan J. F. (1998). A Family of Human Receptors Structurally Related to *Drosophila* Toll. Proc. Natl. Acad. Sci. U.S.A. 95, 588–593. 10.1073/pnas.95.2.588 PubMed Abstract | 10.1073/pnas.95.2.588 | Google Scholar 9435236 PMC18464

[B66] Rohen J. W. Lütjen-Drecoll E. Flügel C. Meyer M. Grierson I. (1993). Ultrastructure of the Trabecular Meshwork 10in Untreated Cases of Primary Open-Angle Glaucoma (POAG). Exp. Eye Res. 56, 683–692. 10.1006/exer.1993.1085 PubMed Abstract | 10.1006/exer.1993.1085 | Google Scholar 8595810

[B67] Rohen J. W. Witmer R. (1972). Electron Microscopic Studies on the Trabecular Meshwork in Glaucoma Simplex. Albr. Graefes Arch. Klin. Ophthalmol. 183, 251–266. 10.1007/bf00496153 10.1007/bf00496153 | Google Scholar 4111808

[B68] Russell P. Johnson M. (2012). Elastic Modulus Determination of Normal and Glaucomatous Human Trabecular Meshwork. Invest. Ophthalmol. Vis. Sci. 53, 117. 10.1167/iovs.11-9314 PubMed Abstract | 10.1167/iovs.11-9314 | Google Scholar 22233636

[B69] Saika S. (2006). TGFβ Pathobiology in the Eye. Lab. Invest. 86, 106–115. 10.1038/labinvest.3700375 PubMed Abstract | 10.1038/labinvest.3700375 | Google Scholar 16341020

[B70] Seki E. De Minicis S. Österreicher C. H. Kluwe J. Osawa Y. Brenner D. A. (2007). TLR4 Enhances TGF-β Signaling and Hepatic Fibrosis. Nat. Med. 13, 1324–1332. 10.1038/nm1663 PubMed Abstract | 10.1038/nm1663 | Google Scholar 17952090

[B71] Sethi A. Jain A. Zode G. S. Wordinger R. J. Clark A. F. (2011a). Role of TGFβ/Smad Signaling in Gremlin Induction of Human Trabecular Meshwork Extracellular Matrix Proteins. Invest. Ophthalmol. Vis. Sci. 52, 5251–5259. 10.1167/iovs.11-7587 PubMed Abstract | 10.1167/iovs.11-7587 | Google Scholar 21642622 PMC3176052

[B72] Sethi A. Mao W. Wordinger R. J. Clark A. F. (2011b). Transforming Growth Factor-β Induces Extracellular Matrix Protein Cross-Linking Lysyl Oxidase (LOX) Genes in Human Trabecular Meshwork Cells. Invest. Ophthalmol. Vis. Sci. 52, 5240–5250. 10.1167/iovs.11-7287 PubMed Abstract | 10.1167/iovs.11-7287 | Google Scholar 21546528 PMC3176072

[B73] Shepard A. R. Millar J. C. Pang I.-H. Jacobson N. Wang W.-H. Clark A. F. (2010). Adenoviral Gene Transfer of Active Human Transforming Growth Factor-Β2 Elevates Intraocular Pressure and Reduces Outflow Facility in Rodent Eyes. Invest. Ophthalmol. Vis. Sci. 51, 2067–2076. 10.1167/iovs.09-4567 PubMed Abstract | 10.1167/iovs.09-4567 | Google Scholar 19959644

[B74] Shibuya E. Meguro A. Ota M. Kashiwagi K. Mabuchi F. Iijima H. (2008). Association of Toll-like Receptor 4 Gene Polymorphisms with Normal Tension Glaucoma. Invest. Ophthalmol. Vis. Sci. 49, 4453–4457. 10.1167/iovs.07-1575 PubMed Abstract | 10.1167/iovs.07-1575 | Google Scholar 18586872

[B75] Stamer W. D. Acott T. S. (2012). Current Understanding of Conventional Outflow Dysfunction in Glaucoma. Curr. Opin. Ophthalmol. 23, 135–143. 10.1097/icu.0b013e32834ff23e PubMed Abstract | 10.1097/icu.0b013e32834ff23e | Google Scholar 22262082 PMC3770936

[B76] Szabo G. Dolganiuc A. Mandrekar P. (2006). Pattern Recognition Receptors: a Contemporary View on Liver Diseases. Hepatology 44, 287–298. 10.1002/hep.21308 PubMed Abstract | 10.1002/hep.21308 | Google Scholar 16871558

[B77] Takano Y. Shi D. Shimizu A. Funayama T. Mashima Y. Yasuda N. (2012). Association of Toll-like Receptor 4 Gene Polymorphisms in Japanese Subjects with Primary Open-Angle, Normal-Tension, and Exfoliation Glaucoma. Am. J. Ophthalmol. 154, 825–832. 10.1016/j.ajo.2012.03.050 PubMed Abstract | 10.1016/j.ajo.2012.03.050 | Google Scholar 22831837

[B78] Tamm E. R. (2009). The Trabecular Meshwork Outflow Pathways: Structural and Functional Aspects. Exp. Eye Res. 88, 648–655. 10.1016/j.exer.2009.02.007 PubMed Abstract | 10.1016/j.exer.2009.02.007 | Google Scholar 19239914

[B79] Tovar-Vidales T. Clark A. F. Wordinger R. J. (2011). Transforming Growth Factor-Beta2 Utilizes the Canonical Smad-Signaling Pathway to Regulate Tissue Transglutaminase Expression in Human Trabecular Meshwork Cells. Exp. Eye Res. 93, 442–451. 10.1016/j.exer.2011.06.011 PubMed Abstract | 10.1016/j.exer.2011.06.011 | Google Scholar 21722634 PMC3389044

[B80] Tripathi R. C. Li J. Chan W. A. Tripathi B. J. (1994). Aqueous Humor in Glaucomatous Eyes Contains an Increased Level of TGF-Β2. Exp. Eye Res. 59, 723–728. 10.1006/exer.1994.1158 PubMed Abstract | 10.1006/exer.1994.1158 | Google Scholar 7698265

[B81] Vahabikashi A. Gelman A. Dong B. Gong L. Cha E. D. K. Schimmel M. (2019). Increased Stiffness and Flow Resistance of the Inner Wall of Schlemm's Canal in Glaucomatous Human Eyes. Proc. Natl. Acad. Sci. U. S. A. 116 (52), 26555–26563. 10.1073/pnas.1911837116 10.1073/pnas.1911837116 | Google Scholar PMC6936716 31806762

[B82] Vittitow J. Borrás T. (2004). Genes Expressed in the Human Trabecular Meshwork during Pressure-Induced Homeostatic Response. J. Cell. Physiol. 201, 126–137. 10.1002/jcp.20030 PubMed Abstract | 10.1002/jcp.20030 | Google Scholar 15281095

[B83] Vranka J. A. Kelley M. J. Acott T. S. Keller K. E. (2015). Extracellular Matrix in the Trabecular Meshwork: Intraocular Pressure Regulation and Dysregulation in Glaucoma. Exp. Eye Res. 133, 112–125. 10.1016/j.exer.2014.07.014 PubMed Abstract | 10.1016/j.exer.2014.07.014 | Google Scholar 25819459 PMC4379427

[B84] Vranka J. A. Staverosky J. A. Reddy A. P. Wilmarth P. A. David L. L. Acott T. S. (2018). Biomechanical Rigidity and Quantitative Proteomics Analysis of Segmental Regions of the Trabecular Meshwork at Physiologic and Elevated Pressures. Invest. Ophthalmol. Vis. Sci. 59, 246–259. 10.1167/iovs.17-22759 PubMed Abstract | 10.1167/iovs.17-22759 | Google Scholar 29340639 PMC5770183

[B85] Weinreb R. N. Aung T. Medeiros F. A. (2014). The Pathophysiology and Treatment of Glaucoma. JAMA 311, 1901–1911. 10.1001/jama.2014.3192 PubMed Abstract | 10.1001/jama.2014.3192 | Google Scholar 24825645 PMC4523637

[B86] Weinreb R. N. Khaw P. T. (2004). Primary Open-Angle Glaucoma. Lancet 363, 1711–1720. 10.1016/s0140-6736(04)16257-0 PubMed Abstract | 10.1016/s0140-6736(04)16257-0 | Google Scholar 15158634

[B87] Welge-Lüssen U. May Ca Eichhorn M. Bloemendal H. Lütjen-Drecoll E. (1999). AlphaB-crystallin in the Trabecular Meshwork Is Inducible by Transforming Growth Factor-Beta. Invest. Ophthalmol. Vis. Sci. 40, 2235–2241. PubMed Abstract | Google Scholar 10476788

[B88] White E. Baralle F. Muro A. (2008). New Insights into Form and Function of Fibronectin Splice Variants. J. Pathol. 216, 1–14. 10.1002/path.2388 PubMed Abstract | 10.1002/path.2388 | Google Scholar 18680111 PMC4630009

[B89] Wordinger R. J. Fleenor D. L. Hellberg P. E. Pang I.-H. Tovar T. O. Zode G. S. (2007). Effects of TGF-Β2, BMP-4, and Gremlin in the Trabecular Meshwork: Implications for Glaucoma. Invest. Ophthalmol. Vis. Sci. 48, 1191–1200. 10.1167/iovs.06-0296 PubMed Abstract | 10.1167/iovs.06-0296 | Google Scholar 17325163

[B90] Yan X. Lin Z. Chen F. Zhao X. Chen H. Ning Y. (2009). Human BAMBI Cooperates with Smad7 to Inhibit Transforming Growth Factor-β Signaling. J. Biol. Chem. 284, 30097–30104. 10.1074/jbc.m109.049304 PubMed Abstract | 10.1074/jbc.m109.049304 | Google Scholar 19758997 PMC2781564

[B91] Yang H. Young D. W. Gusovsky F. Chow J. C. (2000). Cellular Events Mediated by Lipopolysaccharide-Stimulated Toll-like Receptor 4. J. Biol. Chem. 275, 20861–20866. 10.1074/jbc.m002896200 PubMed Abstract | 10.1074/jbc.m002896200 | Google Scholar 10877845

[B92] Yang L. Seki E. (2012). Toll-like Receptors in Liver Fibrosis: Cellular Crosstalk and Mechanisms. Front. Physio. 3, 138. 10.3389/fphys.2012.00138 PubMed Abstract | 10.3389/fphys.2012.00138 | Google Scholar PMC3357552 22661952

[B93] Yang Q.-W. Xiang J. Zhou Y. Zhong Q. Li J. C. (2010). Targeting HMGB1 TLR4 Signaling as a Novel Approach to Treatment of Cerebral Ischemia. Front. Biosci. S2, 1081–1091. 10.2741/s119 PubMed Abstract | 10.2741/s119 | Google Scholar 20515842

[B94] Yang X. Luo C. Cai J. Powell D. W. Yu D. Kuehn M. H. (2011). Neurodegenerative and Inflammatory Pathway Components Linked to TNF-Α/tnfr1 Signaling in the Glaucomatous Human Retina. Invest. Ophthalmol. Vis. Sci. 52, 8442–8454. 10.1167/iovs.11-8152 PubMed Abstract | 10.1167/iovs.11-8152 | Google Scholar 21917936 PMC3208177

[B95] Yarovinsky F. (2014). Innate Immunity to Toxoplasma Gondii Infection. Nat. Rev. Immunol. 14, 109–121. 10.1038/nri3598 PubMed Abstract | 10.1038/nri3598 | Google Scholar 24457485

[B96] Zhang N. Wang J. Li Y. Jiang B. (2021). Prevalence of Primary Open Angle Glaucoma in the Last 20 Years: a Meta-Analysis and Systematic Review. Sci. Rep. 11, 13762. 10.1038/s41598-021-92971-w PubMed Abstract | 10.1038/s41598-021-92971-w | Google Scholar 34215769 PMC8253788

[B97] Zode G. S. Sethi A. Brun-Zinkernagel A. M. Chang I. F. Clark A. F. Wordinger R. J. (2011). Transforming Growth Factor-Β2 Increases Extracellular Matrix Proteins in Optic Nerve Head Cells via Activation of the Smad Signaling Pathway. Mol. Vis. 17, 1745–1758. PubMed Abstract | Google Scholar 21738403 PMC3130727

[B98] Zuliani-Alvarez L. Marzeda A. M. Deligne C. Schwenzer A. Mccann F. E. Marsden B. D. (2017). Mapping Tenascin-C Interaction with Toll-like Receptor 4 Reveals a New Subset of Endogenous Inflammatory Triggers. Nat. Commun. 8, 1595. 10.1038/s41467-017-01718-7 PubMed Abstract | 10.1038/s41467-017-01718-7 | Google Scholar 29150600 PMC5693923

